# Surface defect detection method for discarded mechanical parts under heavy rust coverage

**DOI:** 10.1038/s41598-024-58620-8

**Published:** 2024-04-04

**Authors:** Zelin Zhang, Xinyang Wang, Lei Wang, Xuhui Xia

**Affiliations:** 1 Key Laboratory of Metallurgical Equipment and Control Technology, Ministry of Education, Wuhan University of Science and Technology, Wuhan, 430081 China; 2 Hubei Key Laboratory of Mechanical Transmission and Manufacturing Engineering, Wuhan University of Science and Technology, Wuhan, 430081 China; 3 Precision Manufacturing Institute, Wuhan University of Science and Technology, Wuhan, 430081 China

**Keywords:** Remanufacturing, Defect detection, Deep learning, Heavy rust, Engineering, Mechanical engineering

## Abstract

With a significant number of mechanical products approaching the retirement phase, the batch recycling of discarded mechanical parts necessitates a preliminary assessment of their surface condition. However, the presence of surface rust poses a challenge to defect identification. Therefore, this paper proposes a method for detecting heavily rusted surface defects based on an improved YOLOv8n network. In the Backbone, the C2f-DBB module of re-parameterized deep feature extraction was introduced, and the attention module was designed to improve the accuracy of information extraction. In the Neck part, a Bi-Afpn multiscale feature fusion strategy is designed to facilitate information exchange between features at different scales. Finally, Focal-CIoU is employed as the bounding box loss function to enhance the network’s localization performance and accuracy for defects. Experimentally, it is proved that the improved network in this paper improves the Recall, Precision, and mAP0.5 by 1.2%, 2.1%, and 1.9%, respectively, on the original basis, which is better than other network models.

## Introduction

**Figure 1:**
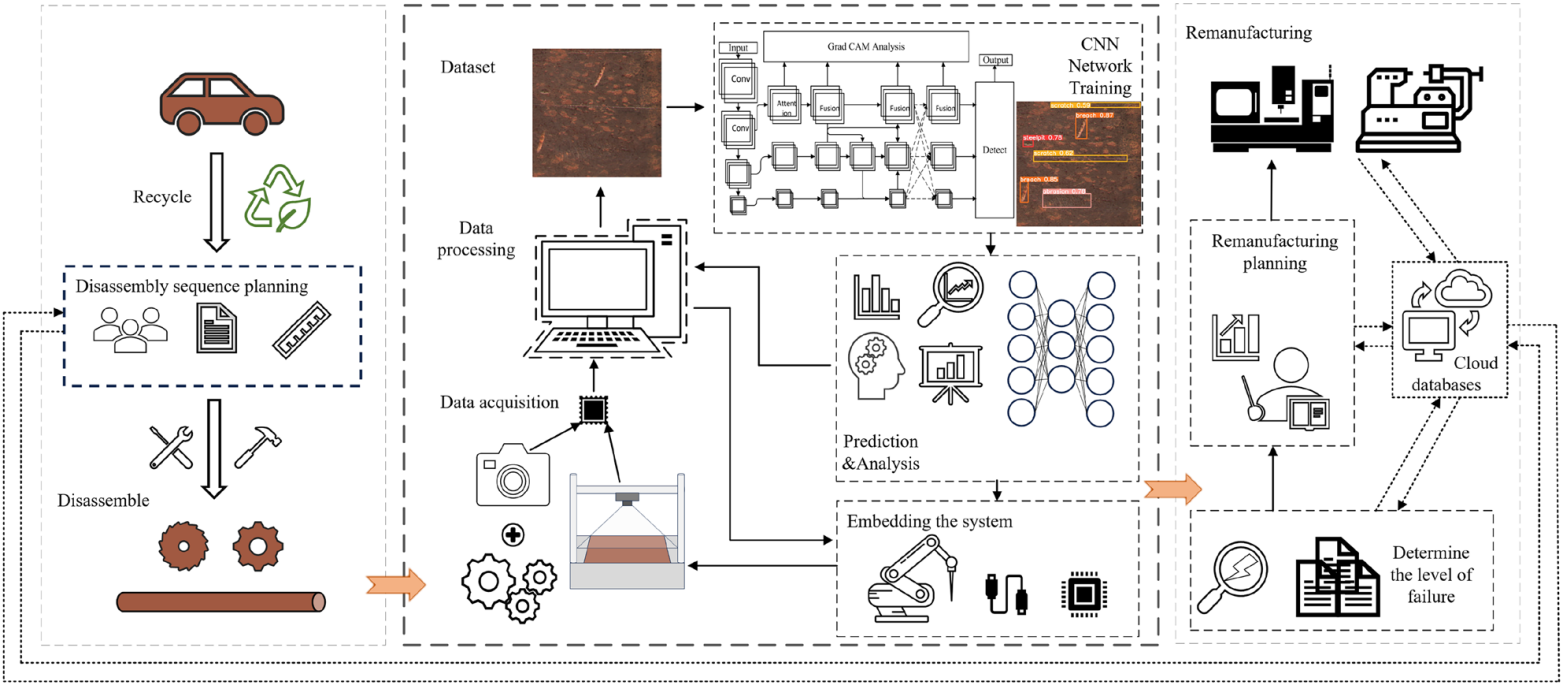
The remanufacturing process of discarded mechanical parts.

With the rapid development of the economy, society, and industrialization, many mechanical products are reaching the peak of retirement. Recycling discarded mechanical products for defect detection and assessment is integral to remanufacturing, as shown in Fig. 1. There are different degrees of defects and rust contamination on the surface of discarded machinery products, so their surface image quality is lower and more difficult to detect. Accurate and fast automatic detection and positioning of surface defects on metal parts covered with rust is crucial for remanufacturing defect level assessment, which can improve efficiency, shorten the production cycle, reduce labor costs, etc. On this basis, the design and planning of remanufacturing processes for discarded parts that have passed the defect level assessment can save resources and energy to a great extent^1^.

The various surface defects on discarded mechanical components and a complex background pose significant challenges to automated detection^2^. When recycling and remanufacturing large quantities of discarded machinery parts, the difficulty of inspection is greatly increased by the fact that the parts themselves have other defects covered by rust and by other factors in storage and transport that result in surface defects of the parts being covered with rust. Heavily rusted parts have increased surface roughness, which can cause blurring of boundaries and internal features of other defects. The rusted areas exhibit random distribution, irregular shapes, and varying shades of color. Additionally, discarded mechanical parts may have other common types of defects on their metal surfaces, such as scratches, breaches, pits, and abrasions. These defects differ in shape, size, and affected area, and do not share a common feature for localization and segmentation.

Conventional methods for removing surface rust and detecting other defects on heavily rusted parts require a lot of human resources. These include removing the rust before detecting defects, but rust removal equipment is expensive, and sandblasting is time-consuming and may damage the metal. Another method is to detect defects directly through the rust layer, but contact detection contaminates the inspection instrument and reduces accuracy. In non-contact inspection methods, image sensor visual inspection is more effective^3^, and other NDT equipment is not good at inspecting complex surfaces, so there is an urgent need for fast and effective defect detection methods for heavy rust cover.

In the task of surface defect detection, machine vision and deep learning for surface defect detection are increasingly used in modern production^4^. In the method of Dubey et al.^5^ features are manually extracted, and the boundaries of rail defects are analyzed for detection. Shi et al.^6^ proposed an improved Sobel operator that reduces edge noise in rail defects for accurate defect localization. Agarwal et al.^7^ introduced a support vector classification scheme, based on process knowledge (PK-MSVM) for defect classification on the surface of hot-rolled steel. Traditional methods such as image processing have a large impact on the affected environment, poor generalization ability, and low efficiency. The detection method of the deep neural network can do automatic detection and automatically frame the location, size, and category of defects. Li et al.^4^ proposed an improved YOLOv4, providing a new approach for defect detection on steel strip surfaces. Tian et al.^2^ designed a DCC-Center-Net model, offering a balance between accuracy and speed for surface defect detection in steel. In the research of Sun et al.^8^ an unsupervised defect detection method for surface defects on aluminum plates with few samples was proposed. However, the early stages of these deep neural network algorithms still require the manual annotation of large datasets. Another disadvantage of CNNs is their weak interpretability, whereas Grad CAM allows for visual interpretation of defects without changing the network structure^9^. The semantic segmentation algorithm also shows the shape of the defects accurately, making it easy to analyze the defects quantitatively and qualitatively, but more fine-grained annotation of the dataset is required. Trained deep learning models are not easy to embed in devices for real-time detection, making automatic detection of surface defects a challenging task^10^. In the task of detecting rusted areas of parts, Lee et al.^11^ examine whether rust areas exist in given color images, Liao et al.^12^ propose a background color-based rust detection technique, and Zhang et al.^13^ introduce a channel attention-based method for metal corrosion detection. In the above study, traditional machine learning-based image processing methods or threshold segmentation algorithms were unable to distinguish other defects in the rusted area, while deep learning-based algorithms also faced difficulties in annotation and the inability to achieve online detection.

In this paper, an efficient detection method for surface defects of heavily rusted parts is proposed based on the YOLOv8n network. Firstly, a dataset of common surface defects such as scratches, breaches, pits, and abrasions under heavy rust coverage is preprocessed, and then the feature extraction network, multi-scale feature fusion network, and bounding box loss function are improved. Then, targets in the dataset are accurately detected and visualized using Grad CAM.

The main contributions of this paper are as follows:

1. In the backbone feature extraction network, the deep feature extraction C2f-DBB module is introduced, which does not affect the inference speed while extracting features in depth. The attention mechanism module is designed to improve the robustness, accuracy, recognition, and characterization of the network.
2. A multi-scale feature fusion strategy, Bi-Afpn, is designed to ensure minimal intermediate layer interference. This strategy promotes feature fusion between non-adjacent layers while minimizing information degradation during global fusion.
3. Focal-CIoU as a bounding box loss function enhances the impact of high-quality samples in the network, thus improving the performance and accuracy of defect localization.

The remaining chapters of this paper are organized as follows: Chapter 2 reviews in detail the work related to the detection and segmentation of surface defects on metal parts, including the detection and segmentation of rusted areas and the detection and segmentation of other defects. Chapter 3 introduces the improvements made in the proposed network, including enhancements to the feature extraction network, multi-scale feature fusion network, and the bounding box loss function. Chapter 4 presents experimental results and analyses, covering ablation experiments, module optimization results, and comparisons with different network models. Chapter 5 summarizes and outlooks the whole paper.

## Related works

### Traditional methods

Currently, most of the inspection methods for surface defects of mechanical parts are based on manual inspection, and the results of manual inspection are largely affected by the following factors such as the intensity of light, the skill level of workers, the degree of defects, and so on. The boundaries and internal features of other defects on the surface of parts covered in heavy rust become blurred, making methods dependent on manual inspection more time-consuming and labor-intensive. In general, to detect other defects under heavy rust coverage, one approach involves removing rust stains from the parts first and then conducting defect detection. Another approach is to detect defects through the rust layer directly. In the task of rust removal, existing laser rust removal equipment achieves significant rust removal effects. Still, it is expensive, requires advanced technical expertise, and may cause slight damage to the surface of the parts. Sandblasting for rust removal is time-consuming and labor-intensive and can also damage the underlying metal. In the direct detection task, when using contact detection instruments, the rust layer can contaminate the detection instruments and reduce the accuracy of defect detection. In non-contact detection methods, traditional non-destructive testing equipment is not adept at detecting complex surfaces, while vision-based detection leveraging image analysis holds distinct advantages.

### Deep learning methods

The emergence of convolutional neural networks (CNNs) has provided powerful methods in the field of computer vision, leading to breakthroughs in various areas such as object detection, semantic segmentation, and image generation. Researchers have made various network improvements to achieve accurate and rapid surface quality detection in mechanical products. Balancing the accuracy and speed of detection models to meet industrial inspection requirements has become a focal point of research.

In the rust detection and segmentation task, the identification of rusted areas is mainly performed by calibrating the rusted areas or distinguishing color features. Atha et al. use a VGG network, the original image is cropped and categorized into rusted or unrusted regions, and then these metal-rusted regions are detected at the patch level^14^. Zhang et al.^13^ proposed a metal rust detection method based on channel attention (CAMCD). This method embeds the SE (Squeeze-and-Excitation) block into a deep residual network, constructing the CAMCD network. The approach is capable of automatically detecting rust areas with multiple distinct levels. Zhao et al.^15^ proposed an improved YOLOv7 network for detecting the rusting of anti-vibration hammer in power systems, using the HSV color model for color space transformation, and then introducing GS-Conv and BiFPN multiscale feature fusion, and both the accuracy and speed of the improved network were improved. Shi et al.^16^ applied VGG-Unet segmentation to a metal surface rust dataset and used Background Data Drop Rate (BDDR) to reduce the number of background images.

In the metal surface defect detection task, a deep learning target detection framework is used as a basis for the calibration of the defective region and the improvement of various aspects of the original network, which in turn leads to the fast and accurate detection of defective targets. Song et al. addressed surface defects on steel plates using variability convolution and background suppression to enhance the recognition accuracy of defects in Fast R-CNN ^17^. However, the image processing speed was limited to 13.1 FPS. Liu et al. utilizing the Fast R-CNN network, introduced a novel RoI pooling method^18^. They combined it with feature pyramid network (FPN) and ResNet-50 for feature extraction and established a new non-maximum suppression method. This approach proved effective for detecting minor defects in aviation generator blades. Zhang et al.^19^ improved the framework of the Efficient-Det network in a single-stage manner based on the two-stage Mask R-CNN. This modification achieved high-precision detection of surface defects in steel materials. The two-stage network can achieve higher detection accuracy but has lower detection efficiency. In contrast, single-stage methods treat object detection as a regression problem, enabling simultaneous position estimation and category classification. Single-stage network structures are more superficial, faster, and highly accurate but may exhibit weaker detection performance for small objects. Kou et al.^20^ improved surface defect detection on steel strips based on the YOLOv3 network, employing an anchor-free feature selection mechanism and an embedded dense convolution block to enhance computational efficiency. However, the capability to detect minor defects was found to be limited. MA et al. conducted lightweight improvements for aluminum strip surface defect detection on the foundation of YOLOv4^21^. This involved techniques such as depth-wise separable convolution, attention mechanisms, lightweight the network neck, and optimizing B-Box loss functions, resulting in a threefold increase in detection speed. Nevertheless, it still falls short of meeting the requirements for real-time industrial detection. Wan et al.^22^ proposed a method for detecting tile defects using an improved version of the YOLOv5s model. Liu et al.^23^ introduced a multi-scale context detection network (MSC-DNet). However, this network lacks a global perspective, which is disadvantageous for detecting minor target defects, making it challenging to effectively apply in practical production scenarios. Zhang et al. developed a defect detection method for steel surfaces based on an improved Retina-Net^10^. This network increased the depth of the feature pyramid network (FPN) to enhance information extraction capabilities, strengthened feature information, and added a detection head specifically for small targets, thereby improving the overall detection capability of the network. However, as the depth of the network increases, more features are fused, leading to a significant increase in the number of parameters. Li et al.^24^ proposed a Mobile-Net-SSD surface defect detection method suitable for quickly detecting typical defects such as breaches, dents, burrs, and abrasions on the sealing surface of containers in filling lines. Wang et al.^25^ proposed a YOLOv5-CD model that combines the coordinate attention mechanism and decoupling head. The algorithm converges faster and better meets the requirements of practical industrial production.

None of the above work reflects the situation where rusted areas and other defects in metal parts coexist, whereas on the surfaces of discarded mechanical parts both conditions coexist, are very common, and the rust is more severe and more difficult to detect. Therefore, building upon previous work, we propose a defect detection method for the surface of discarded mechanical parts under heavy rust coverage based on the YOLOv8n network. In the feature extraction network, we introduce the diverse branch block and attention mechanism to enhance the accuracy of target feature extraction. In the multi-scale feature fusion module, we propose the Bi-Afpn strategy to ensure global fusion with minimal information degradation. Additionally, we introduce the Focal CIoU boundary box loss function to enhance the network’s localization performance and accuracy in detecting defects. Compared to the original YOLOv8n network, the improved network shows improvements in various evaluation metrics, effectively addressing surface defects on discarded mechanical parts under heavy rust coverage, and it meets the requirements for industrial online detection.

## Methodologies

The backbone feature extraction network of YOLOv8 utilizes the C2f module with a more enriched gradient flow, and it adjusts different channel numbers for models of different scales. This involves carefully fine-tuning the model structure, leading to a significant improvement in model performance.

**Figure 2:**
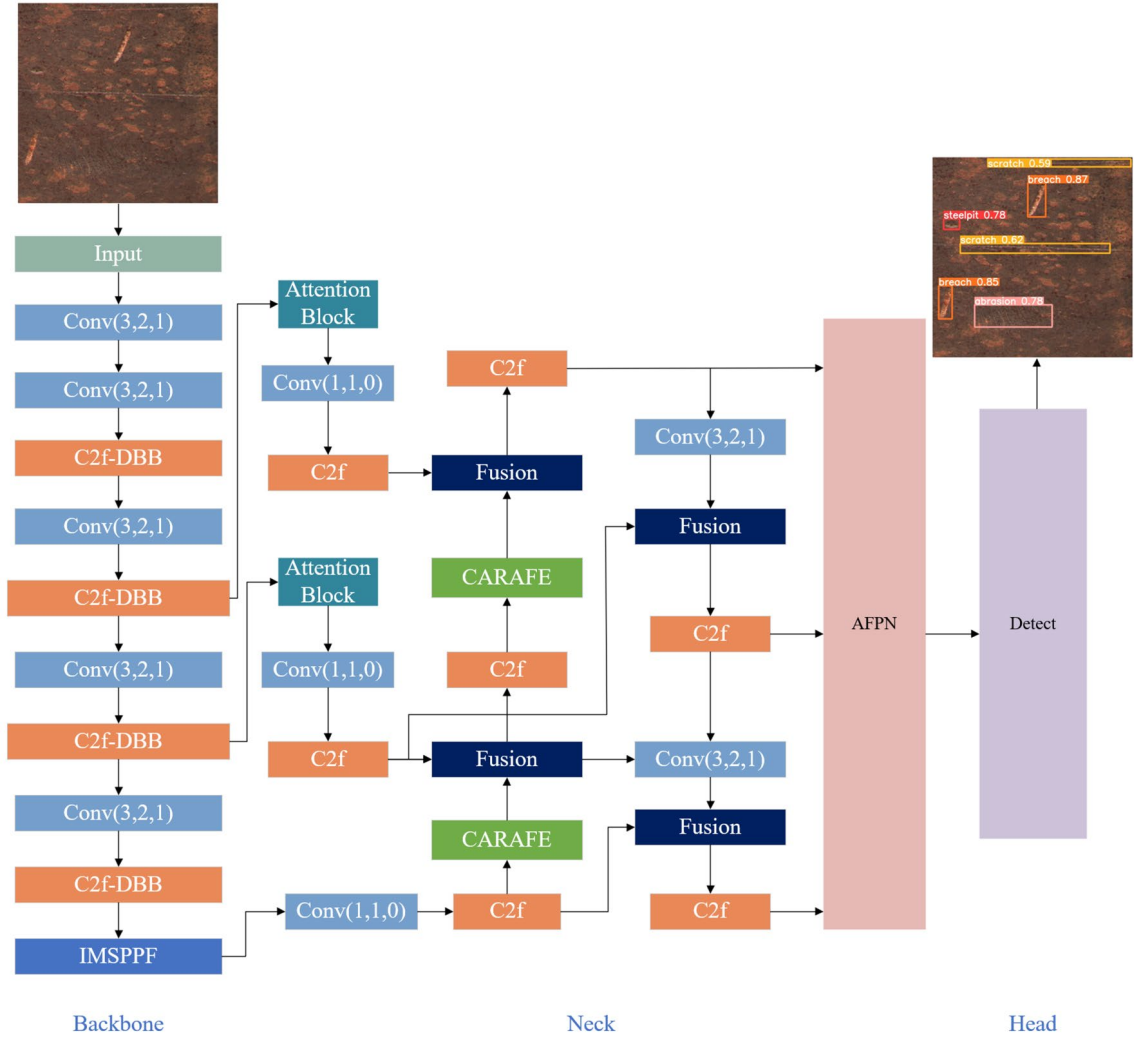
The network model for detecting surface defects on discarded mechanical parts with heavy rust coverage.

Building upon this foundation, the proposed improved network, as illustrated in Fig. 2, includes a feature extraction network that balances model accuracy and speed, a Bi-Afpn multi-scale feature fusion strategy that ensures minimal information degradation for global fusion and promotes direct feature fusion between non-adjacent layers, and a boundary box loss function that enhances the role of high-quality samples. Specifically, the C2f in the backbone network is improved to C2f-DBB with a reparameterization mechanism, which not only avoids an increase in model parameters during the inference process but also enhances the model’s average precision. The attention mechanism module has been designed, and it has been integrated into the IMSPPF module replacing the SPPF module, which provides a more accurate identification of defects under the reparameterization overlay in the global channel and in spatial coordinates improves accuracy. Subsequently, the feature maps are input into the Bi-Afpn multi-scale feature fusion strategy and finally fed into the detection head.

### Backbone feature extraction network

#### C2f-DBB module

**Figure 3:**
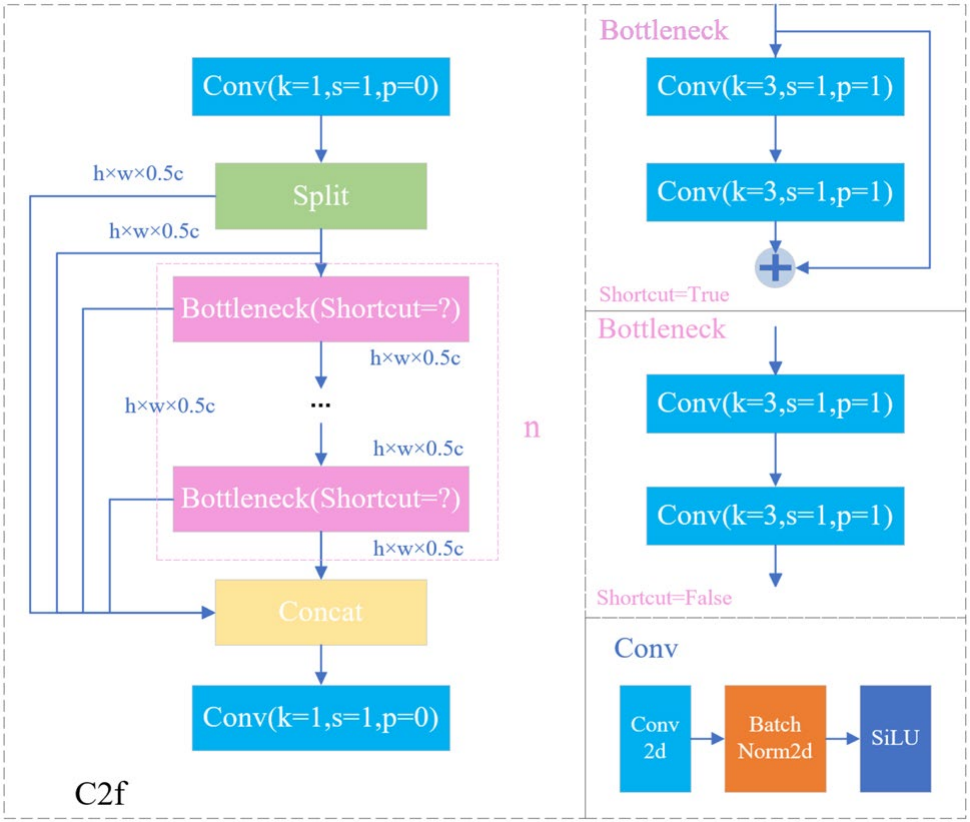
C2f module.

**Figure 4:**
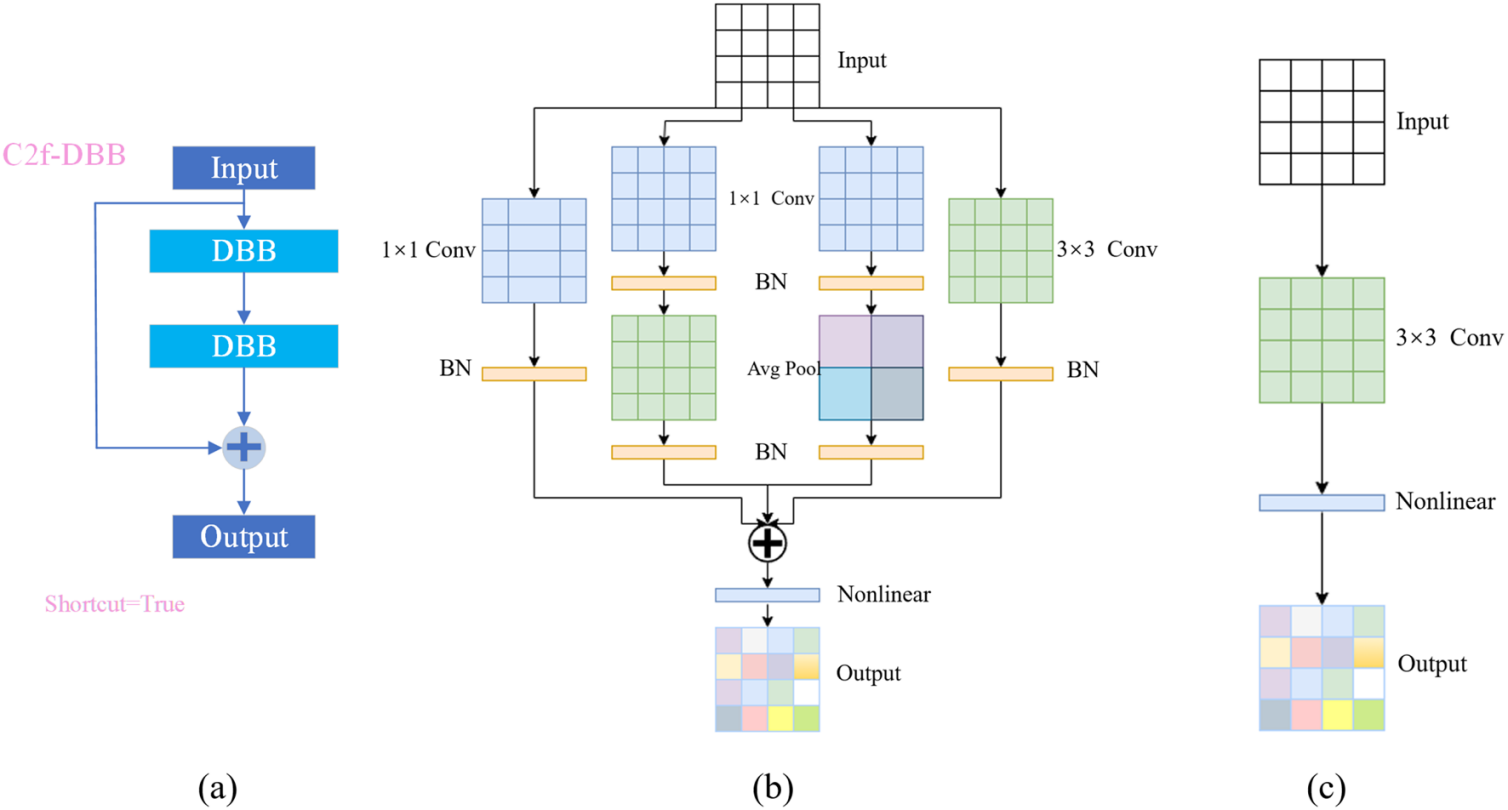
C2f-DBB module. (**a**) is the C2f feature extraction module embedded in BDD, (**b**) is the training process of DBB, and (**c**) is the inference process of DBB.

YOLOv8n’s C2f module consists of two 1 × 1 convolutions, a Split operation, several Bottleneck blocks, and a concatenate operation. Specifically, when the feature map passes through the C2f module, as shown in Fig. 3, 1 × 1 convolution is first performed, and then the feature map is separated. One-half of the convolved feature map is input to the Bottleneck block, and the remaining concatenate the parts. Half of the feature map of the part that enters the Bottleneck enters the next Bottleneck block. Half of it is spliced until the feature map obtained after n Bottlenecks is spliced with the above-mentioned part that needs to be short-spliced. Then, it undergoes a 1 × 1 convolution for information integration. Where Bottleneck exists in C2f. Shortcut is true and false two. To balance the speed and accuracy, this paper refers to the idea of a multiparameter mechanism of multi-branch structure and embeds DBB^26^ into the Bottleneck of C2f, as in Fig. 4. DBB involves six conversion modes, namely BN layer conversion, convolution branch addition conversion, convolution sequence conversion, deep connection conversion, average pooling conversion, and multi-scale convolution conversion. Each branch of DBB can be converted into a convolution with the same inference time as the structure and rule conversion layers, but the former has stronger representation power. When training the model, DBB will input the input feature map into four routes at the same time, namely the ordinary 3 × 3 convolution route, the 1 × 1 convolution route, the average pooling route, and the convolution sequence route, and finally add the four routes. During model inference, DBB will optimize the deep feature extraction part into a convolution and a nonlinear layer, which not only does not lose the inference speed but also greatly improves the inference accuracy of the model.

#### Attention mechanism module

Due to its flexible structural characteristics, the attention mechanism is not only conducive to learning more discriminative feature representations but can also be easily inserted into the backbone structure of CNN. Therefore, the attention mechanism has attracted widespread attention in the field of computer vision research. In recent years, various attention mechanism modules have emerged one after another and developed rapidly, such as SE^27^, CBAM^28^, GAM^29^, SimAM^30^, and so on; these attention modules can be integrated seamlessly into any CNN architectures, easy to operate, and show fantastic performance improvements in object detection tasks.

**Figure 5:**
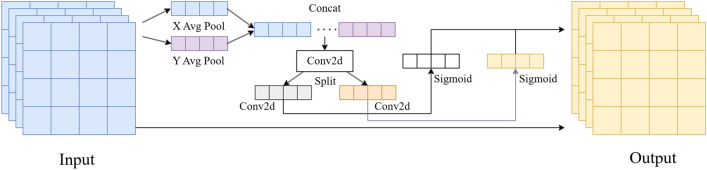
Coordinate attention mechanism.

The coordinate attention mechanism^31^ embeds position information into channel attention and aggregates features along two spatial directions, respectively, as shown in Fig. 5. This allows capturing long-range dependencies in one spatial direction while maintaining precise location information in another.

Given input X, encode each channel along the horizontal axis with a spatial range of (H, 1) and along the vertical axis with a spatial range of (1, W) using pooling kernels. The output can be represented as Eqs. (1) and (2):

1 $$\begin{array}{c}{Z}_{c}^{h}\left(h\right)=\frac{1}{W}\sum_{0\le i<W}{X}_{c}\left(h,i\right)\end{array}$$

2 $$\begin{array}{c}{Z}_{c}^{w}\left(w\right)=\frac{1}{h}\sum_{0\le i<h}{X}_{c}\left(w,i\right)\end{array}$$

After adaptive average pooling and concatenation, the feature maps are then sent to the shared 1 × 1 convolutional transformation function, denoted as ${f}^{1\times 1}$. The output is shown in Eq. (3).

3 $$\begin{array}{c}F=\delta \left({f}_{1}\left(\left[{z}^{h},{z}^{w}\right]\right)\right)\end{array}$$

As shown in the above expression: $\left[{z}^{h},{z}^{w}\right]$ represents the concatenation operation along the spatial dimension, $\delta$ is the non-linear activation function, $F \in {\mathbb{R}}^{{\frac{c}{r} \times \left( {H + W} \right)}}$ is the intermediate feature map encoding spatial information in the horizontal and vertical directions, and $r$ is the compression ratio. Subsequently, the feature map is split along the spatial dimension into two tensors, $F^{h} \in {\mathbb{R}}^{{\frac{c}{r} \times H}}$ and $F^{w} \in {\mathbb{R}}^{{\frac{c}{r} \times W}}$. Next, ${F}^{h}$ and ${F}^{w}$ are transformed into tensors with the same number of channels as the input X, as stated in Eqs. (4) and (5).

4 $$\begin{array}{c}{g}^{h}=\sigma \left({f}_{h}\left({F}^{h}\right)\right)\end{array}$$

5 $$\begin{array}{c}{g}^{w}=\sigma \left({f}_{w}\left({F}^{w}\right)\right)\end{array}$$

$\sigma$ represents the sigmoid function, $F$ is the convolutional transformation, and then the outputs ${g}^{h}$ and ${g}^{w}$ are respectively unfolded and weighted with attention, the output of y is represented as Eq. (6):

6 $$\begin{array}{c}{y}_{c}\left(i,j\right)={x}_{c}\left(i,j\right)\times {g}_{c}^{h}\left(i\right)\times {g}_{c}^{w}\left(j\right)\end{array}$$

**Figure 6:**
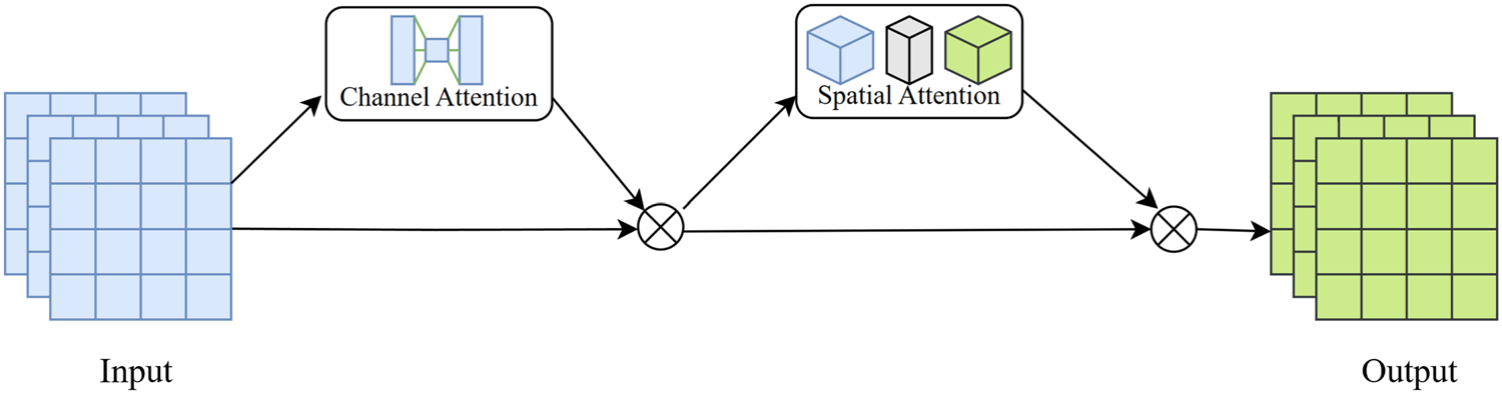
Global attention mechanism.

Similar to the CBAM attention mechanism, GAM (Global Attention Mechanism) follows a two-step process of channel attention applied to the feature map first, then followed by spatial attention, as shown in Fig. 6. However, the CBAM attention mechanism neglects the interaction between channels and spatial dimensions, resulting in the loss of cross-dimensional information. In contrast, the GAM possesses the ability to reduce information loss and enhance the interaction of features across global dimensions. It places greater emphasis on feature details and has achieved superior performance compared to CBAM. The formula is as Eqs. (7) and (8):

7 $$\begin{array}{c}{F}^{\prime}={M}_{c}\left(F\right)\otimes F\end{array}$$

8 $$\begin{array}{c}{F}^{{\prime}{\prime}}={M}_{s}\left({F}^{\prime}\right)\otimes {F}^{\prime}\end{array}$$

The input feature map $F\in {\mathbb{R}}^{C\times H\times W}$ undergoes a channel attention mechanism initially. The channel attention submodule employs a 3D arrangement to preserve cross-dimensional information in ${M}_{C}\left(F\right)$. The element-wise operation between ${M}_{C}\left(F\right)$ and the original feature map $F$ yields a refined feature map ${F}^{\prime}$. Subsequently, the refined feature ${F}^{\prime}$ map undergoes a spatial attention mechanism, transforming it into a feature ${M}_{s}\in {\mathbb{R}}^{C\times H\times W}$. The final feature map ${F}^{{\prime}{\prime}}$ is obtained through element-wise operation with ${F}^{\prime}$.

The channel attention module first reorganizes the input feature map $f$, then connects it through a MLP (Multilayer Perceptron), and finally reverts the arrangement. The calculation process is as Eq. (9):

9 $$\begin{aligned} Mc\left( F \right) = & \sigma \left( {MLP\left( {Resize\left( F \right)} \right)} \right) \\ = & \sigma \left( {W_{1} \left( {W_{0} \left( F \right)} \right)} \right) \\ \end{aligned}$$

$\sigma$ is the Relu activate function,${W}_{0}\in {\mathbb{R}}^{C\times C/r}$, ${W}_{1}\in {\mathbb{R}}^{C/r\times C}$, $r$ is the compression ratio, ${W}_{0}$ and ​${W}_{1}$ represent the weights associated with the two inputs in the MLP.

The spatial attention module takes the processed feature map $f^{\prime}$ from the channel attention module as input. It then undergoes a convolutional layer to generate a two-dimensional spatial attention map, the formula as Eq. (10).

10 $$\begin{array}{c}{M}_{s}\left({F}^{\prime}\right)={\sigma }_{1}\left({f}^{7\times 7}\left(\sigma \left({f}^{7\times 7}\left({F}^{\prime}\right)\right)\right)\right)\end{array}$$

$\sigma$ is the Relu activate function, ${\sigma }_{1}$ is Sigmoid function, ${F}^{7\times 7}$ represent the convolutional kernel with the size of 7 × 7.

**Figure 7:**
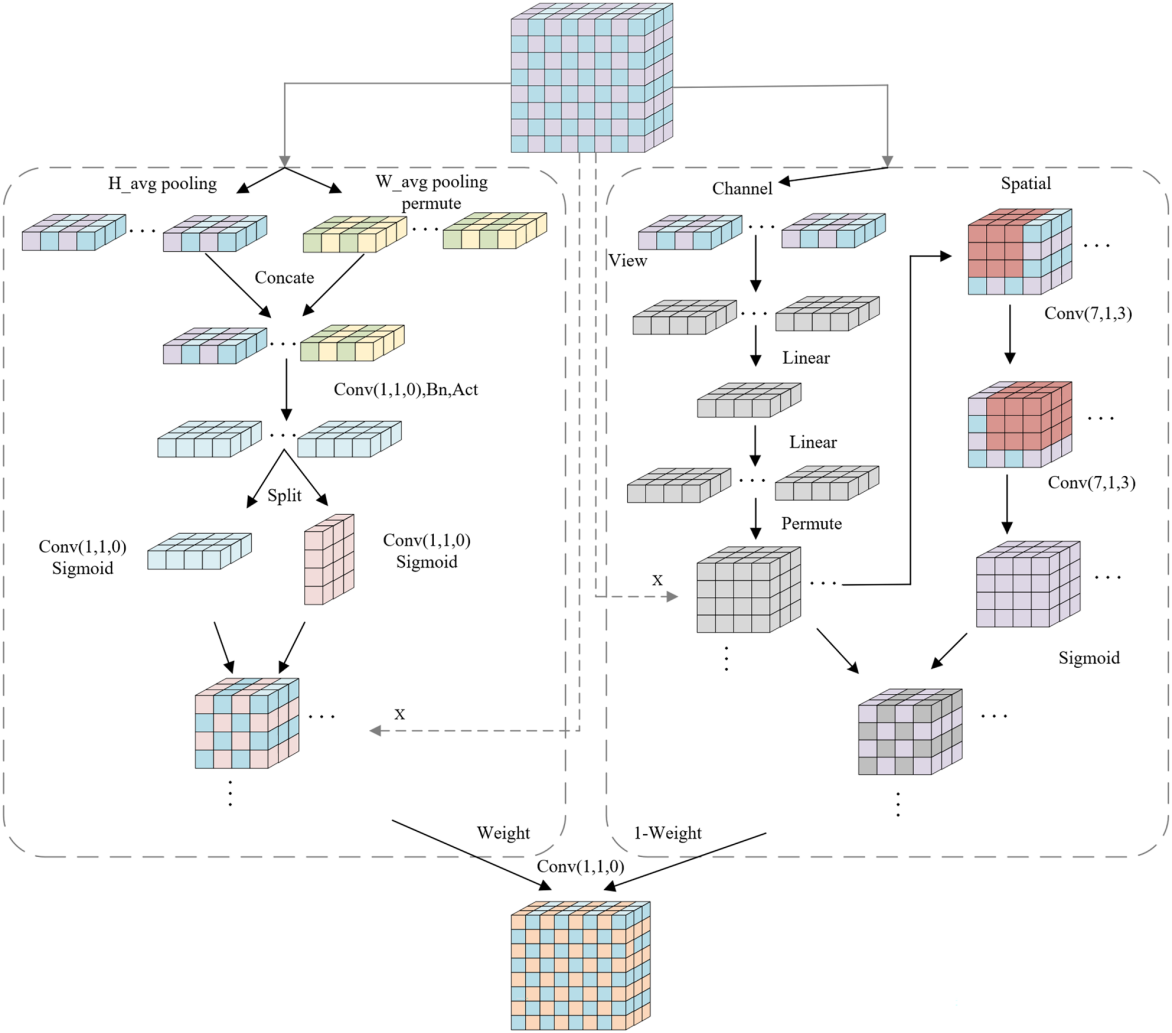
Our attention mechanisms.

The Coordinate attention mechanism focuses on the interaction of channel information and the localization position information but lacks detailed information in the global dimension. On the other hand, the GAM attention mechanism emphasizes the detailed information in the global dimension and the interaction of channel information but lacks the effect of position information. Therefore, this paper combines these two attention mechanisms, assigns weights to each, and combines the outputs of the two attention mechanisms with weighting. Subsequently, a 1 × 1 convolution is applied to enhance learning capabilities while maintaining feature smoothness, as shown in Fig. 7 and expressed by Eq. (11).

11 $$\begin{array}{c}Output={f}^{1\times 1}\left(\mu \left({y}_{c}\left(i,j\right)\right)+\left(1-\mu \right){M}_{s}\left({F}^{\prime}\right)\right)\end{array}$$

where ${y}_{c}\left(i,j\right)$ represents the output of the Coordinate attention mechanism, ${M}_{s}\left({F}^{\mathrm{^{\prime}}}\right)$ represents the output of the GAM, $\mu$ denotes the applied weight, and ${f}^{1\times 1}$ represents a 1 × 1 convolutional kernel.

**Figure 8:**
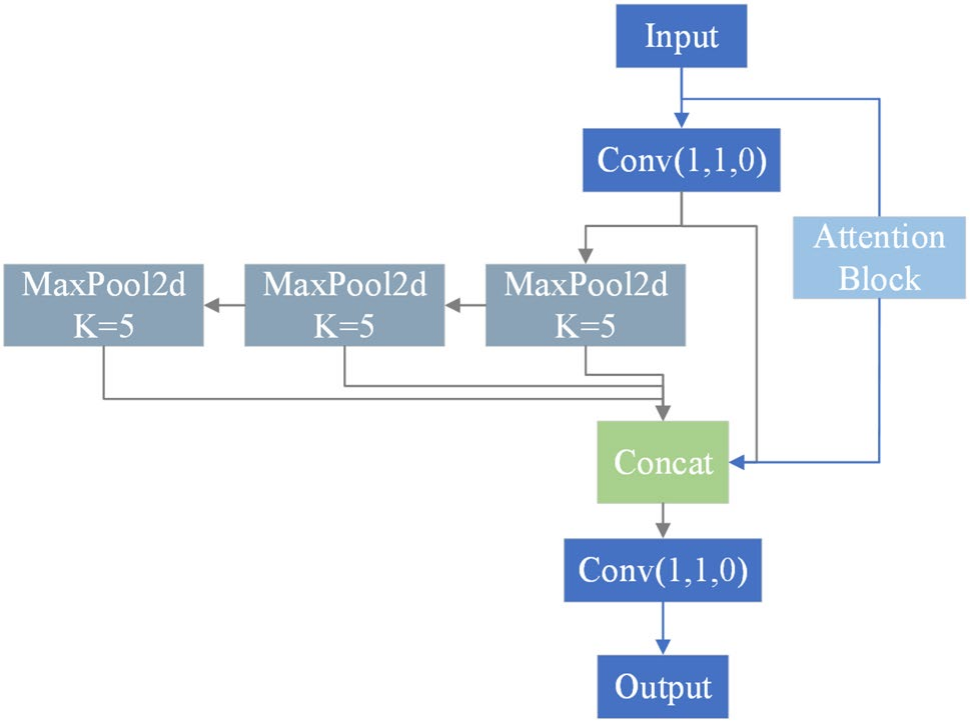
IMSPPF module.

In this paper, the attention mechanism is embedded in SPPF, and the IMSPPF structure is designed as in Fig. 8. The role of SPP is to increase the receptive field, allowing the algorithm to adapt to different-resolution images. It achieves this by using max-pooling to obtain different receptive fields. SPP and SPPF have similar effects but differ slightly in structure. In SPP, the convolutional kernels in Maxpool2d are (5, 9, 13), while in SPPF, the convolutional kernels in Maxpool2d are all 5. SPP adopts a parallel structure, whereas SPPF adopts a serial structure, improving processing speed. SSPF contains rich abstract semantic information, and adding attention mechanisms to this part of the feature maps allows for global channel and spatial focus on the target.

### Multi-scale feature fusion module

**Figure 9:**
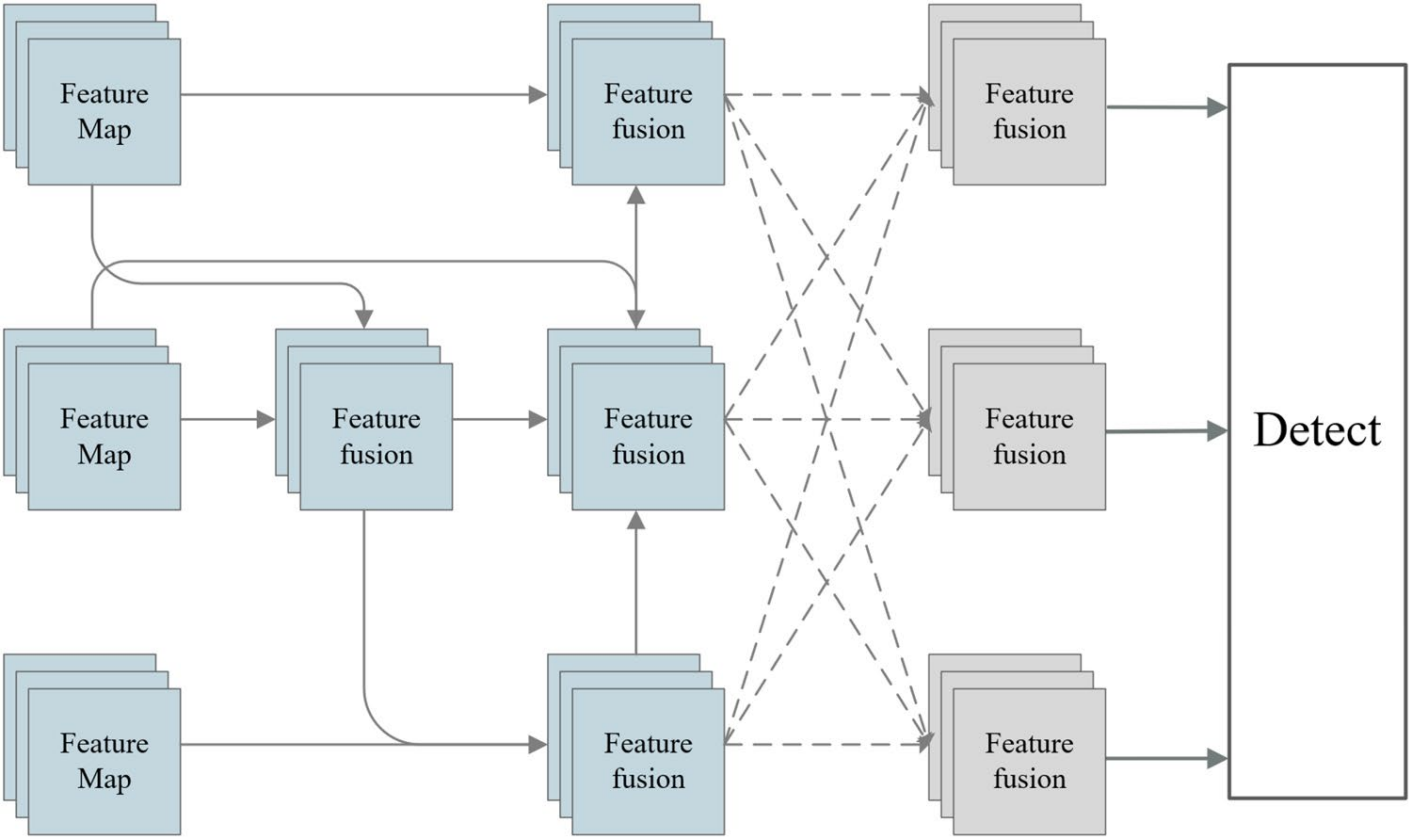
Bi-Afpn structure plan (Dashed lines represent adaptive spatial feature fusion).

Object detection models typically incorporate multi-scale feature fusion to address the issue of scale variations in targets. In this condition, higher-level features need to propagate through multiple intermediate scales and interact with features at these intermediate scales to fuse with lower-level features. During this propagation and interaction process, semantic information of high-level features may be lost or degraded. Meanwhile, the bottom-up path poses the opposite problem, where detailed information of the bottom-level features may be lost or degraded during propagation and interaction. Therefore, the original multi-scale feature fusion module of YOLOv8n is improved, which makes a better effect on the dataset of this paper. Our Approach is inspired by two feature fusion strategies, Bifpn^32^ and AFPN^33^, and designs a Bi-Afpn multi-scale feature fusion module, as illustrated in Fig. 9. Specifically, the fusion method of Bifpn is used instead of the AFPN fusion for the first two layers of feature maps. In Bifpn, fusion is applied to the P3, P4, and P5 layers of the backbone feature extraction network. This involves both bottom-up and top-down fusion methods, along with skip connections. This design ensures minimal loss of high-level semantic information by reducing the number of intermediate layers and also minimizes information degradation during global fusion. The CARAFE up-sampling method is employed to replace traditional up-sampling, reducing computational complexity. The features processed by Bifpn are input into AFPN, which has only three input layers. This promotes direct feature fusion between non-adjacent layers, preventing the loss or degradation of feature information during transmission and interaction. Additionally, the feature maps processed by Bifpn have mitigated the issue of large semantic information gaps between feature maps of different scales, which could lead to suboptimal fusion effects.

Traditional feature fusion often simply concatenates feature maps without distinguishing between different feature maps being simultaneously superimposed. However, since different input feature maps have different resolutions, Bifpn employs a weighted feature fusion mechanism, combining bidirectional cross-scale connections and fast normalization fusion. The computation is as Eqs. (12) and (13).

12 $$\begin{array}{c}{P}_{n}^{td}=Conv\left(\frac{{w}_{1}\cdot {P}_{n}^{in}+{w}_{2}\cdot Resize\left({P}_{n+1}^{in}\right)}{{w}_{1}+{w}_{2}+\epsilon }\right)\end{array}$$

13 $$\begin{array}{c}{P}_{n}^{out}=Conv\left(\frac{{w}_{1}^{,}\cdot {P}_{n}^{in}+{w}_{2}^{,}\cdot {P}_{n}^{td}+{w}_{3}^{\prime}\cdot Resize\left({P}_{n-1}^{out}\right)}{{w}_{1}^{,}+{w}_{2}^{,}+{w}_{3}^{,}+\epsilon }\right)\end{array}$$

where ${P}_{n}^{td}$ is the intermediate feature of the nth layer in the bottom-up, ${P}_{n}^{out}$ is the output feature of the nth layer in the top-down path, ${P}_{n}^{in}$ is the input of the nth layer, the Resize operation involves both images up-sampling and down-sampling, and $w$ represents the learned parameters.

The core of AFPN is the adaptive spatial feature fusion strategy (ASFF), which is augmented with four residual units’ learning features. Each residual unit consists of two 3 × 3 convolutions. ASFF allocates different spatial weights to features at different layers during the multi-level feature fusion process, enhancing the importance of critical layers and mitigating the impact of conflicting information from different objects. This paper integrates features from three levels based on the YOLOv8n framework. Let ${x}_{ij}^{n\to l}$ represent the feature vector from layer $n$ to layer $l$ at position $(i, j)$. The feature vector ${y}_{ij}^{l}$​ is obtained through adaptive spatial fusion of multi-level features. The computation is defined as a linear combination of feature vectors ${x}_{ij}^{1\to l}$, ${x}_{ij}^{2\to l}$, and ${x}_{ij}^{3\to l}$​, as Eq. (14):

14 $$\begin{array}{c}{y}_{ij}^{l}={\alpha }_{ij}^{l}\cdot {x}_{ij}^{1\to l}+{\beta }_{ij}^{l}\cdot {x}_{ij}^{2\to l}+{\gamma }_{ij}^{l}\cdot {x}_{ij}^{3\to l}\end{array}$$

where ${\alpha }_{ij}^{l}$​, ${\beta }_{ij}^{l}$​, and ${\gamma }_{ij}^{l}$ represent the spatial weights of the three levels of features at layer $l$, subject to the constraint ${\alpha }_{ij}^{l}+{\beta }_{ij}^{l}+{\gamma }_{ij}^{l}=1$.

Firstly, the feature maps with different numbers of channels in the input are normalized, bringing uniformity to the channel numbers and preparing for subsequent fusion. In the feature fusion process, CARAFE is employed for image up-sampling, and a 3 × 3 convolutional kernel is used for down-sampling. The feature map sizes for feature fusion are 20 × 20, 40 × 40, and 80 × 80, respectively. During feature fusion, the 20 × 20 feature map is up-sampled and fused with the 40 × 40 feature map. Subsequently, up-sampling and channel compression are applied, and the processed image is then fused with the 80 × 80 image. Finally, channel compression is performed again. For the intermediate layer, the 20 × 20 feature map is up-sampled, fused with the processed 40 × 40 feature map, and channel compression is applied, followed by up-sampling. Then, it is fused with the 80 × 80 feature map, and channel compression is performed. At this point, the resulting 80 × 80 feature map is input into AFPN. Next, the 80 × 80 feature map is down-sampled, fused with the intermediate layer, and channel compression is applied, resulting in a 40 × 40 feature map input to AFPN. Finally, the 40 × 40 feature map is down-sampled fused with the 20 × 20 feature map, channel compression is performed, and the resulting 20 × 20 feature map is input to AFPN. In AFPN, the three sizes of feature maps are interleaved, and four residual units are added to learn features. Finally, the three-size feature maps are input into the detection head.

### Bounding box loss function

Bounding Box Regression (BBR) is a crucial step in determining the performance of object localization, where the bounding box loss function plays an extremely important role in the convergence of the target bounding boxes. The expression for IoU (Intersection over Union) is as Eq. (15):

15 $$\begin{array}{c}{L}_{IOU}=1-\frac{\left|A\cap B\right|}{\left|A\cup B\right|}\end{array}$$

IoU is the ratio of the intersection to the union between the predicted box and the ground truth box, where A and B are any two shapes (or volumes). IoU exhibits good properties such as non-negativity, symmetry, and triangle inequality. In comparison to loss functions like L1/L2, IoU possesses characteristics like scale invariance, making it more effective in reflecting the differences between predicted boxes and ground truth boxes. However, when the predicted box and the ground truth box do not intersect, IoU fails to accurately reflect the tight connection between these two boxes and cannot provide information about the degree of overlap between them.

CIoU^34^ considers three geometric factors: overlapping area Eq. (17), normalized center point distance Eq. (18), and aspect ratio Eq. (19).

16 $$\begin{array}{c}L=S\left(B,{B}_{gt}\right)+D\left(B,{B}_{gt}\right)+V\left(B, {B}_{gt}\right)\end{array}$$

17 $$\begin{array}{c}S=1-IoU\end{array}$$

18 $$\begin{array}{c}D=\frac{{\rho }^{2}\left(P,{P}^{gt}\right)}{{c}^{2}}\end{array}$$

19 $$\begin{array}{c}V={\frac{4}{{\pi }^{2}}(arctan\frac{{w}^{gt}}{{h}^{gt}}-arctan\frac{w}{h})}^{2}\end{array}$$

In the formulas, S, D, and V respectively represent the overlapping area, distance, and aspect ratio. Therefore, the complete CIoU Loss is represented by the following expression, Eq. (20):

20 $$\begin{array}{c}{L}_{CIoU}=1-IoU+\frac{{\rho }^{2}\left(P,{P}^{gt}\right)}{{c}^{2}}+\alpha \left[{\frac{4}{{\pi }^{2}}(arctan\frac{{w}^{gt}}{{h}^{gt}}-arctan\frac{w}{h})}^{2}\right]\end{array}$$

In the formula, a variable α is introduced, represented as Eq. (21):

21 $$\begin{array}{*{20}c} {\alpha = \left\{ {\begin{array}{*{20}c} {0,} & {if\;IoU < 0.5} \\ {\frac{V}{{\left( {1 - IoU} \right) + V}},} & {if\;IoU \ge 0.5} \\ \end{array} } \right.} \\ \end{array}$$

When IoU < 0.5, the CIoU loss degrades to the DIoU loss. This implies that when the predicted box and the ground truth box do not match well, the consistency of the aspect ratio is not as crucial. However, when IoU ≥ 0.5, the consistency of the aspect ratio becomes necessary.

During the network training process, outliers can generate excessively large gradients, which are harmful to the training process^35^. Therefore, it is crucial to make high-quality samples contribute more to the gradient during the network training process. The role of Focal L1 loss is to increase the contribution of high-quality examples. It is expressed as Eq. (22):

22 $$\begin{array}{c}{L}_{Foca{l}_{CIOU}}={IOU}^{\gamma }{L}_{CIOU}\end{array}$$

In the formula, γ is a parameter that controls the degree of suppression of outliers, with a default value of γ = 0.5.

## Experiments

### Dataset

The data for this experiment consists of images captured by an industrial CCD linear array camera of discarded mechanical parts covered by heavy rust, resulting in four types of defects: scratches, breaches, pits, and abrasions. The captured images, initially sized at 8192 × 8192 pixels, were cropped to 640 × 640. Labelimg was used for annotating the cropped images. To ensure dataset diversity, data augmentation techniques such as flipping and rotating by 90° were applied to the collected images. The final dataset comprises 1905 images, 8485 targets, and corresponding labels. Furthermore, 70% of the dataset is used for training, 10% for validation, and 20% for testing. The number of defects for each class is shown in Table 1.

**Table 1:** Type and number of defects in the data set.

Types of defects	Steel pits	Abrasions	Breaches	Scratches
Numbers	2526	1896	2326	1737

The hardware configuration for the experiments in this paper is shown in Table 2.

**Table 2:** Network framework parameters and hardware configuration.

	Parameter name	Value
Model parameters	Input Size	640 × 640
	Number of iterations	250
	Batch size	4
	Optimizer	SGD
	Learning rate	1 ${e}^{-2}$
	Momentum	0.937
	Confidence	0.5
	IoU	0.6
Environment	GPU	NVIDIA GTX 1070
	CPU	lntel(R) Core(TM)i7-7700
	System	Windows 10
	Platform	Python3.8

### Results and analysis

#### Evaluation metrics

In object detection tasks, the mean Average Precision (mAP) is commonly used as an evaluation metric to measure the performance of detection models. Its calculation is related to Precision (P) and Recall (R) and represents the mean of the Average Precision (AP) across different classes. The formulas for Precision (P) and Recall (R) are as Eqs. (23) and (24):

23 $$\begin{array}{c}P=\frac{{N}_{tp}}{{N}_{tp}+{N}_{fp}}\end{array}$$

24 $$\begin{array}{c}R=\frac{{N}_{tp}}{{N}_{tp}+{N}_{fn}}\end{array}$$

In the formulas, ${N}_{tp}$ represents the number of true positives (correctly classified as positive), ${N}_{fp}$ represents the number of false positives (incorrectly classified as positive), and ${N}_{fn}$ represents the number of false negatives (incorrectly classified as negative).

The formula for calculating the mean Average Precision (mAP), which is the mean of Average Precisions (AP) across all classes, is as Eq. (25):

25 $$\begin{array}{c}AP={\int }_{0}^{1}PRdR\end{array}$$

The formula for calculating the mean Average Precision (mAP), which is the mean of Average Precisions (AP) across all classes, is as Eq. (26):

26 $$\begin{array}{c}mAP=\frac{1}{M}\sum_{k=1}^{M}AP\left(k\right)\end{array}$$

F1 score is the harmonic mean of precision and recall, providing a balanced response to the precision and recall of a model. Its calculation formula is as Eq. (27):

27 $$\begin{array}{c}F1=2\times \frac{R\times P}{R+P}\end{array}$$

#### Ablation experiment

**Figure 10:**
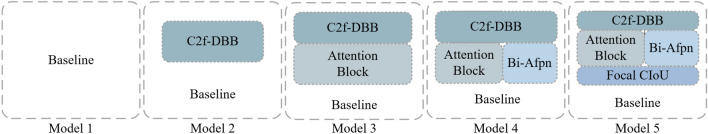
Individual models of ablation experiments.

To assess the effectiveness of the proposed improvements in various modules of the network, ablation experiments were conducted using YOLOv8n as the baseline. The goal was to observe changes in metrics by adding modules. The models for each ablation experiment are shown in Fig. 10, and the corresponding metrics for each model are presented in Table 3. The experimental results indicate that compared to Model 1, the addition of the reparameterized deep extraction module in Model 2 led to improvements of 0.4% in Precision, 0.9% in mAP, and 0.1% in F1 score. However, Recall decreased by 0.3%. It can be inferred that the inclusion of this module has a significant positive impact on the model’s performance. Model 3, built upon Model 2 by incorporating attention mechanisms and IMSPPF, demonstrated improvements compared to Model 2. Specifically, Recall increased by 0.9%, Precision by 0.4%, mAP by 0.6%, and F1 score by 0.6%. This module contributed to a slight overall performance enhancement in the model. Model 4, with the addition of the Bi-Afpn multi-scale feature fusion module, demonstrated improvements compared to Model 3. Specifically, Precision increased by 0.5%, mAP by 0.3%, and F1 score by 0.2%. However, Recall decreased by 0.1%. Model 5, incorporating Focal-CIoU as an improved bounding box loss function, demonstrated the best performance among the five models. Compared to Model 4, Recall increased by 0.7%, Precision by 1.2%, mAP by 0.1%, and F1 score by 0.8%, with a significant improvement in precision. Overall, the improved network outperformed the original network across various evaluation metrics. Recall increased by 1.2%, Precision by 2.1%, mAP by 1.9%, and F1 score by 1.7%.

**Table 3:** Ablation experiment.

Model	Methods					Recall (%)	Precision (%)	mAP0.5 (%)	F1 (%)
	Baseline	DBB	Attention	Bi-Afpn	Focal-CIoU				
Model 1	√					89.5	87.3	91.7	88.3
Model 2	√	√				89.2	87.7	92.6	88.4
Model 3	√	√	√			90.1	88.1	93.2	89.0
Model 4	√	√	√	√		90.0	88.6	93.5	89.2
Model 5	√	√	√	√	√	**90.7**	**89.4**	**93.6**	**90.0**

The optimal value in each metric is bolded.

#### Comparison of module optimization results

**Figure 11:**
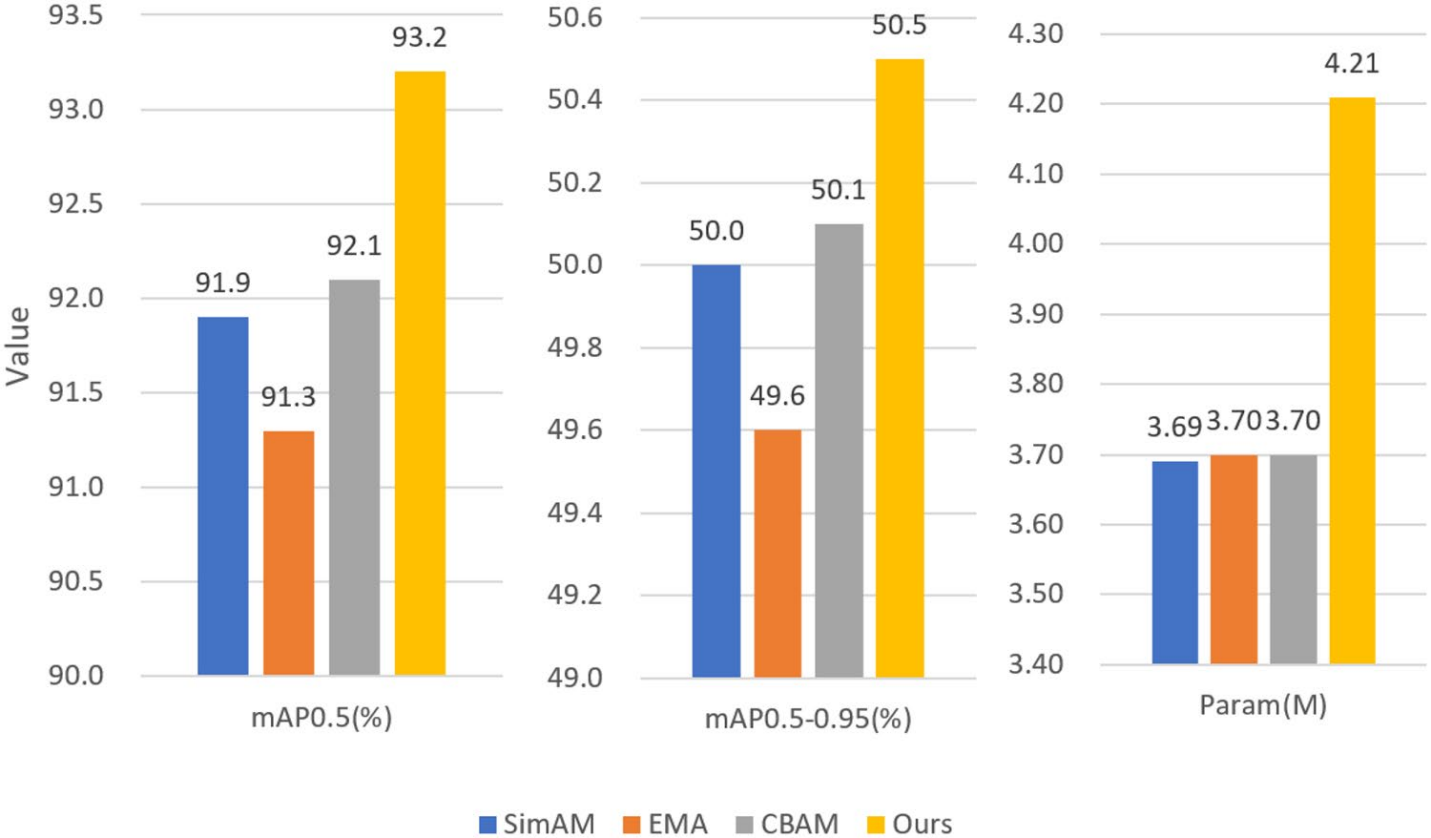
Comparison of attention module indicators.

The paper conducted comparative experiments on attention mechanism modules, using model 2 as the baseline. From Fig. 11, it can be observed that in the parameter-free SimAM attention mechanism, the parameter count is the least, and the mAP is relatively high. In the case of comparable parameter counts, CBAM provides higher mAP compared to EMA. The method proposed in this paper has the highest parameter count but also achieves satisfactory results.

**Figure 12:**
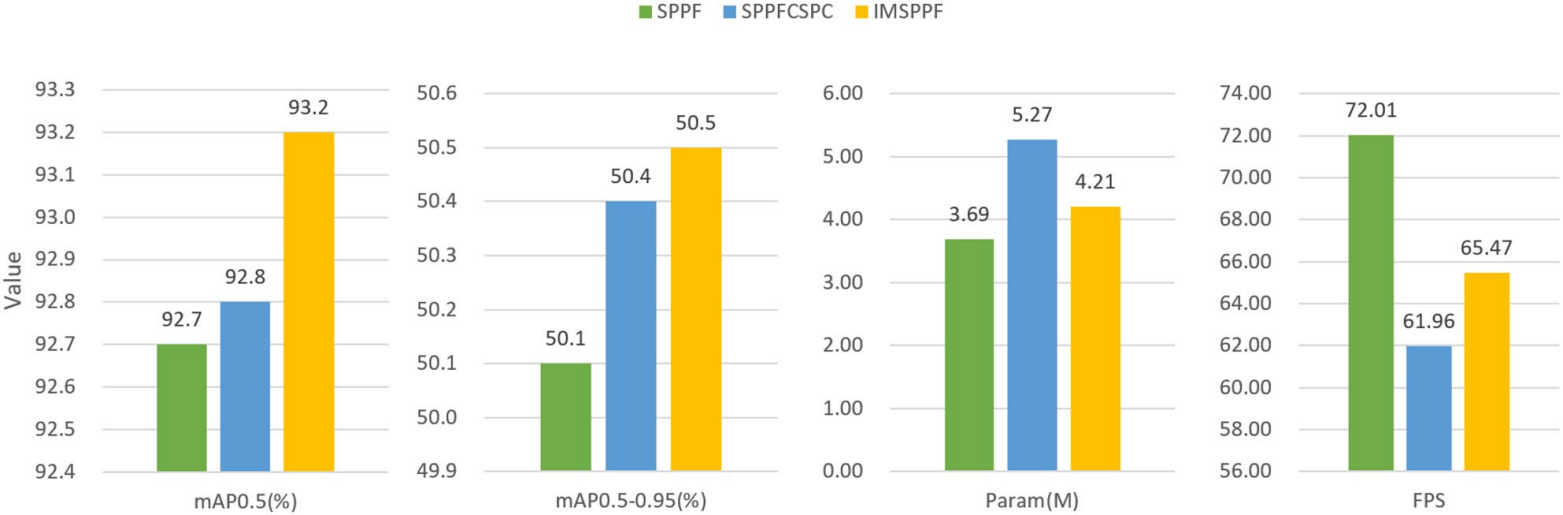
Feature pyramid module comparison.

The paper conducted comparative experiments on the IMSPPF module against the SPPFCSPC module and SPPF module. As shown in Fig. 12, the improved spatial pyramid IMSPPF achieves higher mAP with fewer parameters compared to SPPFCSPC, effectively balancing the precision and speed of the network model.

This paper conducted comparative experiments on the fusion weights in the attention mechanism. From Table 4, it can be observed that the model performs the best when the weight is set to 0.3.

**Table 4:** Attention model metrics with different weights.

Base	μ	mAP0.5 (%)	mAP0.5–0.95 (%)	Recall (%)	Precision (%)
Model2	0	92.7	49.6	**90.3**	86.6
Model2	0.3	**93.2**	**50.5**	90.1	88.1
Model2	0.5	92.9	50.3	90.2	87.8
Model2	0.7	92.7	49.9	**90.3**	87.2
Model2	1	92.6	49.4	87.7	**88.3**

The optimal value in each metric is bolded.

The attention mechanism proposed in this paper is also effective on public datasets. Table 5 shows that on the NEU-DET dataset, with the optimizer set to Adam, Epoch set to 350, and attention weight set to 0.5, the model performs the best. In particular, Precision increased by 9.1%, mAP0.5 increased by 0.8%, and mAP0.5–0.95 increased by 1.1%.

**Table 5:** Performance of our attention mechanism in the NEU-DET dataset.

Base	Precision (%)	Recall (%)	mAp0.5 (%)	mAp0.5–0.95 (%)
v8n	73.0	**68.7**	71.9	37.5
V8n + Att(0.3)	66.9	67.2	69.9	37.2
V8n + Att(0.5)	**82.1**	67.8	**72.7**	**38.6**

The optimal value in each metric is bolded.

**Figure 13:**
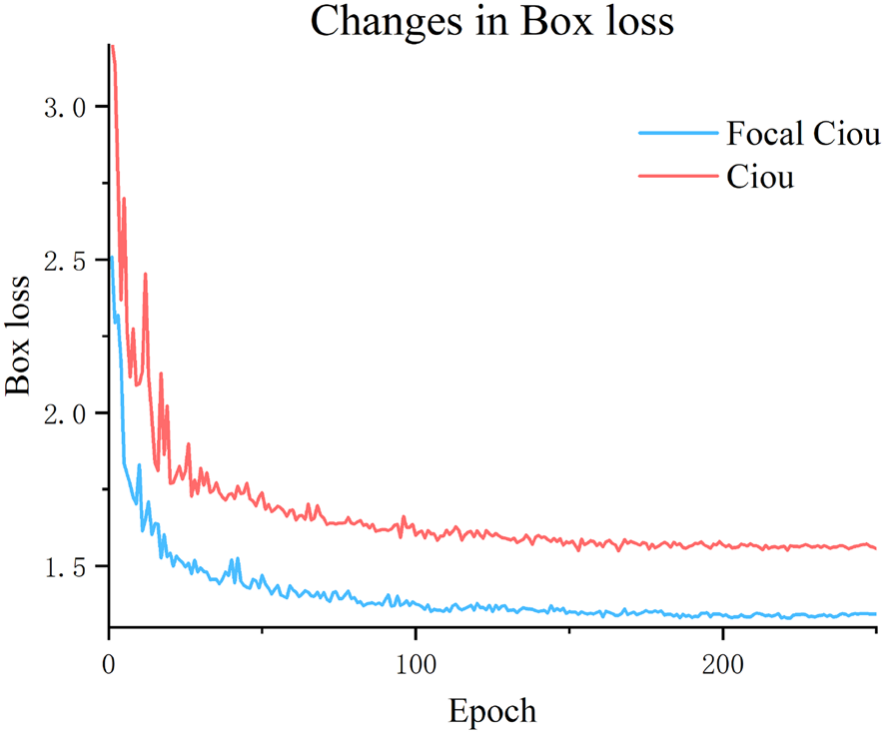
Bounding box loss function comparison.

The bounding box loss functions used in this paper are compared with the original loss functions, as shown in Fig. 13.

#### Comparison of different network models

To further validate the effectiveness of the proposed improved network, it was compared with single-stage networks, two-stage networks, and anchor-free networks using a consistent dataset and hardware configuration. The single-stage networks include YOLOv8n, YOLOv7, YOLOv5n, YOLOv5m, SSD and EfficientDet. The two-stage network is Faster R-CNN, while the anchor-free network is Centernet. The metrics for comparison include Recall, Precision, mAP, F1 score, Parameters, GFLOPs, and FPS. From Table 6, it can be seen that the network proposed in this paper is ahead of other networks in terms of recall, mAP0.5, and F1 value, and is comparable to YOLOv5n in terms of the metrics of Precision. Since the YOLOv5n model has fewer parameters and GFLOPs than the one proposed in this paper, and the complexity of the model operations is low, YOLOv5 is ahead of the other networks in the three metrics of the number of parameters, GFLOPs, and FPS.

**Table 6:** Comparison of different network model metrics.

Series	Model		Recall (%)	Precision (%)	mAP0.5 (%)	F1 (%)	Params	GFLOPs	FPS
One-stage	YOLOv8n		89.5	87.3	91.7	88.3	3.01 M	8.2	76.92
	YOLOv7		85.7	84.6	88.9	87.8	37.62 M	106.5	12.67
	YOLOv5n		89.1	**89.4**	89.9	89.2	**1.76 M**	**4.1**	**84.03**
	YOLOv5m		89.6	89.3	91	89.4	20.86 M	47.9	32.89
	SSD		85.6	54.2	76.8	66.4	26.29 M	62.74	37.05
	EfficientDet	D0	66.1	88.9	75.8	75.8	3.87 M	5.23	8.56
		D3	67.5	88.7	76.4	76.7	12.02 M	50.70	4.51
Two-stage	Faster Rcnn		89.7	47.2	77.5	61.8	137.09 M	370.21	6.92
Anchor free	Centernet		56.4	87.1	86.2	68.4	32.67 M	70.22	22.08
One-stage	Ours		**90.7**	**89.4**	**93.6**	**90.0**	3.72 M	12.6	45.26

The optimal value in each metric is bolded.

**Figure 14:**
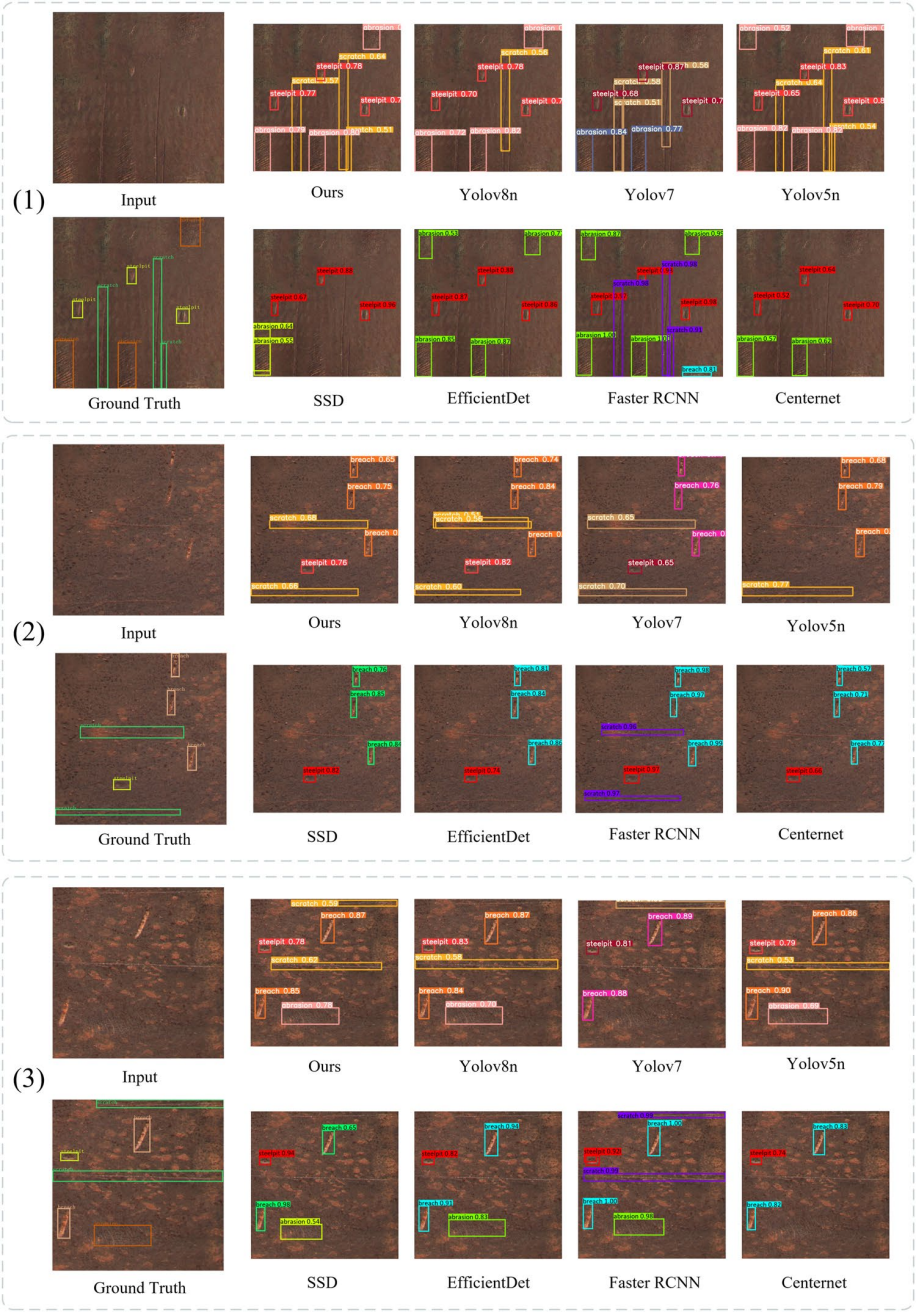
Comparison of the detection effect of different network models.

The processing capabilities of different network models on the rusty part defect dataset are shown in Table 7 and Fig. 14. The comparison involves YOLOv8n, YOLOv7, YOLOv5n, YOLOv5m, EfficientDet D0/D3, SSD, Faster R-CNN, and Centernet network models. It can be observed that the proposed improved network model in this paper demonstrates significant detection performance for all defects in the dataset. The average precision indicators for pits, abrasions, and scratches are particularly notable, achieving 96.9%, 89.3%, and 90.5%, respectively. For breaches, YOLOv5m has the highest average precision, outperforming the proposed model by 0.1%. However, the proposed model surpasses YOLOv5m in average precision for other defects.

**Table 7:** mAP0.5 for defect types with different model outputs.

Series	Model		mAP 0.5 for all defect types			
			Steel pits (%)	Abrasions (%)	Breaches (%)	Scratches (%)
One-stage	YOLOv8n		96.2	84.8	95.7	**90.5**
	YOLOv7		96.4	82.6	93.8	81.3
	YOLOv5n		95.8	84.1	95.2	84.3
	YOLOv5m		96	84.6	**96.4**	87.2
	SSD		79.5	77.4	82.5	67.4
	EfficientDet	D0	94.1	76.6	94.1	38.4
		D3	92.6	80.6	94.1	36.9
Two-stage	Faster Rcnn		73.1	80.1	72.2	84.8
Anchor free	Centernet		91.8	79.5	89.4	84.3
One-stage	Ours		**96.9**	**89.3**	96.3	**90.5**

The optimal value in each metric is bolded.

**Figure 15:**
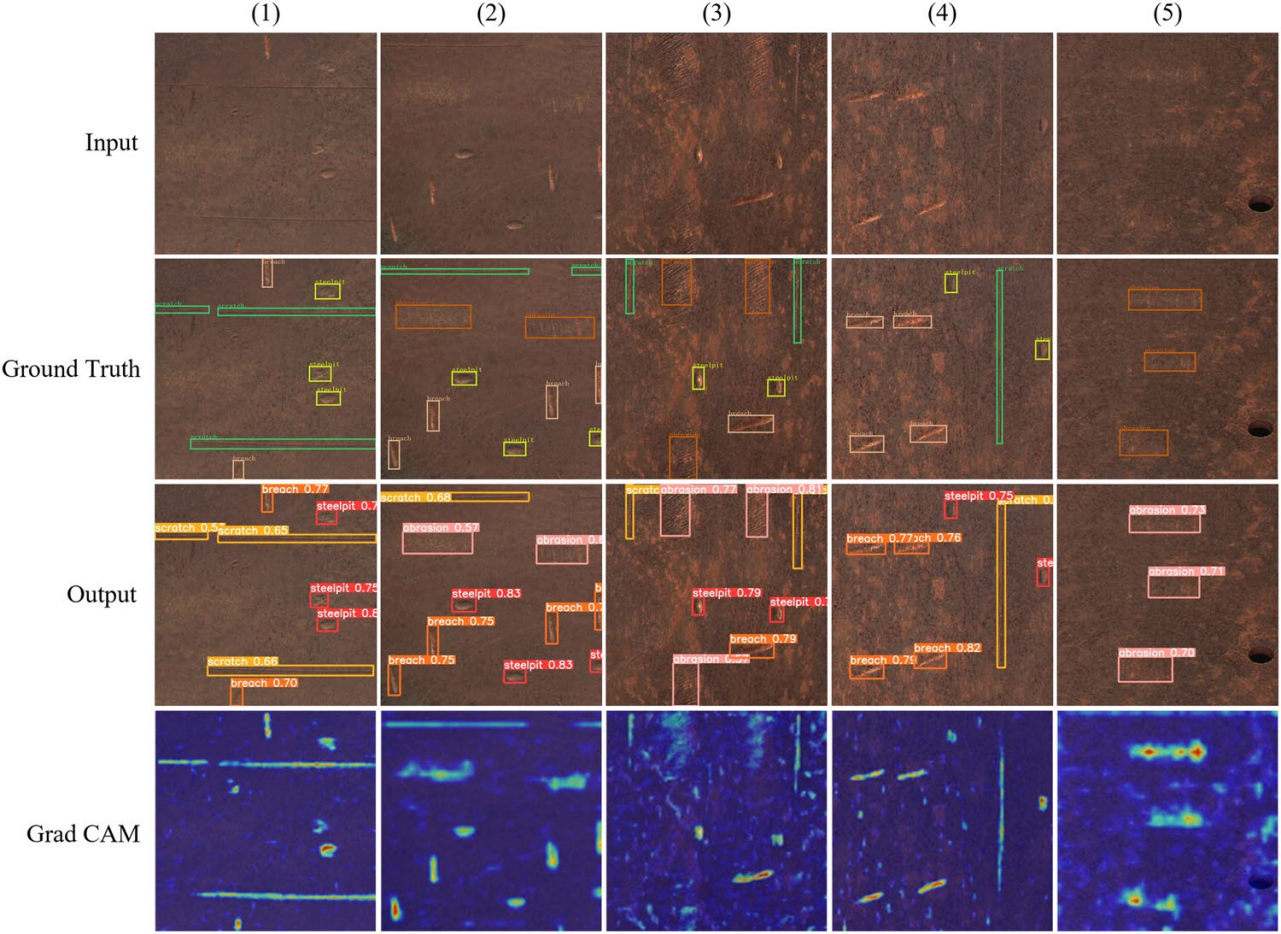
Our improvement in the effectiveness of network detection.

From Table 6 and Fig. 14, it can be seen that the method proposed in this paper is superior to the other models, while in terms of both rapidity and model size, the method proposed in this paper is not as good as YOLOv5n’s network. From the mAP0.5 of the various types of defects in Table 7, it can be seen that the performance of YOLOv5m in the breaches of this target is better than in this paper, which is the most direct reason that the model parameter number and the amount of operation of YOLOv5m is larger compared with this paper, and the number of layers of the network is deeper, which will make the model fit the target better, especially in the feature fusion stage, the deeper the layers are, the more semantic information it can have, which plays an important role in the detection of small and medium-sized targets. Secondly, the morphological features of breaches are more recognizable than those of pits, so YOLOv5m has better breaches detection. The detection effect of this paper with Grad-CAM is shown in Fig. 15.

## Conclusion

In this paper, an improved YOLOv8n network-based model is proposed for solving the problem of surface defect detection of discarded mechanical parts under heavy rust cover. The use of deep feature extraction in the backbone network improves the detection accuracy without degrading the inference speed. An attention mechanism is introduced and its generalization ability is demonstrated on a public dataset, introduced a multi-scale feature fusion module for Bi-Afpn that facilitates feature fusion while reducing information degradation. The IoU loss function of Focal CIoU is employed to enhance the role of high-quality samples in the network and improve the performance and accuracy of defect localization. Overall, the proposed model improves the Recall by 1.2%, the Precision by 2.1%, and the mAP0.5 by 1.9% over the original.

Although this paper provides a good detection method to solve the surface defects of discarded mechanical parts under heavy rust coverage, this method sacrifices most of the inference speed of the model. In future work we will work on image-based methods to restore the original appearance of defects covered by rust; secondly, we will continue to explore model pruning experiments and combine them with this paper’s method to achieve a balance of lightweight, detection precision and inference speed, and finally embed the improved network into a real-world inspection device.

## Data Availability

The data that support the findings of this study are available from the author, Zelin Zhang, zhangzelin@wust.edu.cn. upon reasonable request.

## References

[1] Du X, Shen Y, Zhao W, Chen J, Liu R, Wei Y (2023). Wire arc additive manufacturing from the perspective of remanufacturing: A review of data processing. J. Manuf. Process..

[2] Tian R, Jia M (2022). DCC-CenterNet: A rapid detection method for steel surface defects. Measurement.

[3] Mordia R, Verma AK (2022). Visual techniques for defects detection in steel products: A comparative study. Eng. Fail. Anal..

[4] Li M, Wang H, Wan Z (2022). Surface defect detection of steel strips based on improved YOLOv4. Comput. Electr. Eng..

[5] Dubey AK, Jaffery ZA (2016). Maximally stable extremal region marking-based railway track surface defect sensing. IEEE Sens. J.

[6] Shi T, Kong J, Wang X, Liu Z, Zheng G (2016). Improved Sobel algorithm for defect detection of rail surfaces with enhanced efficiency and accuracy. J. Cent. South Univ..

[7] Agarwal K, Shivpuri R, Zhu Y, Chang TS, Huang H (2011). Process knowledge based multi-class support vector classification (PK-MSVM) approach for surface defects in hot rolling. Expert Syst. Appl..

[8] Sun Q, Xu K, Liu H, Wang J (2023). Unsupervised surface defect detection of aluminum sheets with combined bright-field and dark-field illumination. Opt. Lasers Eng..

[9] Xiao G, Zhu B, Zhang Y, Gao H (2023). FCSNet: A quantitative explanation method for surface scratch defects during belt grinding based on deep learning. Comput. Ind..

[10] Hu X, Yang J, Jiang F, Hussain A, Dashtipour K, Gogate M (2023). Steel surface defect detection based on self-supervised contrastive representation learning with matching metric. Appl. Soft Comput..

[11] Lee S, Chang LM, Skibniewski M (2006). Automated recognition of surface defects using digital color image processing. Autom. Constr..

[12] Liao KW, Lee YT (2016). Detection of rust defects on steel bridge coatings via digital image recognition. Autom. Constr..

[13] Zhang S, Deng X, Lu Y, Hong S, Kong Z, Peng Y, Luo Y (2021). A channel attention based deep neural network for automatic metallic corrosion detection. J. Build. Eng.

[14] Atha DJ, Jahanshahi MR (2017). Evaluation of deep learning approaches based on convolutional neural networks for corrosion detection. Struct. Health Monit..

[15] Zhao Z, Guo G, Zhang L, Li Y (2023). A new anti-vibration hammer rust detection algorithm based on improved YOLOv7. Energy Rep..

[16] Shi J, Dang J, Cui M, Zuo R, Shimizu K, Tsunoda A, Suzuki Y (2021). Improvement of damage segmentation based on pixel-level data balance using VGG-Unet. Appl. Sci..

[17] Song C, Chen J, Lu Z, Li F, Liu Y (2023). Steel surface defect detection via deformable convolution and background suppression. IEEE Trans. Instrum. Meas..

[18] Liu Y, Wu D, Liang J, Wang H (2023). Aeroengine blade surface defect detection system based on improved faster RCNN. Int. J. Intell. Syst..

[19] Zhang, C., Yu, B., & Wang, W. Steel surface defect detection based on improved MASK RCNN. In *2022 IEEE 8th International Conference on Computer and Communications (ICCC)*. 10.1109/iccc56324.2022.10065774 (2021).

[20] Kou X, Liu S, Cheng K, Qian Y (2021). Development of a YOLO-V3-based model for detecting defects on steel strip surface. Measurement.

[21] Ma Z, Li Y, Huang M, Huang Q, Cheng J, Tang S (2022). A lightweight detector based on attention mechanism for aluminum strip surface defect detection. Comput. Ind..

[22] Wan G, Fang H, Wang D, Yan J, Xie B (2022). Ceramic tile surface defect detection based on deep learning. Ceram. Int..

[23] Liu R, Huang M, Gao Z, Cao Z, Cao P (2023). MSC-DNet: An efficient detector with multi-scale context for defect detection on strip steel surface. Measurement.

[24] Li Y, Huang H, Xie Q, Yao L, Chen Q (2018). Research on a surface defect detection algorithm based on MobileNet-SSD. Appl. Sci..

[25] Wang B, Wang M, Yang J, Luo H (2023). YOLOv5-CD: Strip steel surface defect detection method based on coordinate attention and a decoupled head. Meas. Sens..

[26] Ding, X., Zhang, X., Han, J., Ding, G. Diverse branch block: Building a convolution as an inception-like unit. In *IEEE Conference on Computer Vision and Pattern Recognition*. 10.48550/arXiv.2103.13425 (2021).

[27] Hu, J., Shen, L., Sun, G. Squeeze-and-excitation networks. In *IEEE Conference on Computer Vision and Pattern Recognition*. 10.48550/arXiv.1709.01507 (2017).

[28] Woo, S., Park, J., Lee, J. Y. & Kweon, I.S. CBAM: Convolutional block attention module. In *European Conference on Computer Vision*. 10.48550/arXiv.1807.06521 (2018).

[29] Liu. Y., Shao, Z., & Hoffmann, N. Global attention mechanism: Retain information to enhance channel-spatial interactions. In *IEEE/CVF Conference on Computer Vision and Pattern Recognition*. 10.48550/arXiv.2112.05561 (2021).

[30] Yang, L., Zhang, R. Y., Li, L. & Xie, X. SimAM: A simple, parameter-free attention module for convolutional neural networks. In *International Conference on Machine Learning*. https://proceedings.mlr.press/v139/yang21o.html. (2021).

[31] Hou, Q., Zhou, D. & Feng, J. Coordinate attention for efficient mobile network design. In *IEEE Conference on Computer Vision and Pattern Recognition*. 10.1109/CVPR46437.2021.01350 (2021).

[32] Tan, M., Pang, R. & Le, Q. V. EfficientDet: Scalable and efficient object detection. In *IEEE Conference on Computer Vision and Pattern Recognition*. 10.48550/arXiv.1911.09070 (2020).

[33] Yang, G., Lei, J., Zhu, Z., Cheng, S., Feng, Z. & Liang, R. AFPN: Asymptotic feature pyramid network for object detection. 10.48550/arXiv.2306.15988 (2023).

[34] Zheng Z, Wang P, Ren D, Liu W, Ye R, Hu Q, Zuo W (2022). Enhancing geometric factors in model learning and inference for object detection and instance segmentation. IEEE Trans. Cybern..

[35] Zhang YF, Ren W, Zhang Z, Jia Z, Wang L, Tan T (2022). Focal and efficient IOU loss for accurate bounding box regression. Neurocomputing.

